# Staurosporine from *Streptomyces sanyensis* activates Programmed Cell Death in *Acanthamoeba* via the mitochondrial pathway and presents low *in vitro* cytotoxicity levels in a macrophage cell line

**DOI:** 10.1038/s41598-019-48261-7

**Published:** 2019-08-12

**Authors:** Luis Cartuche, Ines Sifaoui, Darío Cruz, María Reyes-Batlle, Atteneri López-Arencibia, José Javier Fernández, Ana R. Díaz-Marrero, José E. Piñero, Jacob Lorenzo-Morales

**Affiliations:** 1 Instituto Universitario de Enfermedades Tropicales y Salud Pública de Canarias, Universidad de la Laguna, Avda. Astrofísico Fco. Sánchez s/n, 38206 La Laguna, Santa Cruz de Tenerife, Spain; 2 Instituto Universitario de Bio-Orgánica Antonio González (IUBO AG), Centro de Investigaciones Biomédicas de Canarias (CIBICAN), Universidad de La Laguna (ULL), Avda. Astrofísico Fco. Sánchez 2, 38206 La Laguna, Tenerife, Spain; 3 Departamento de Química Orgánica, Universidad de La Laguna (ULL), Avda. Astrofísico Fco. Sánchez s/n, 38206 La Laguna, Tenerife, Spain; 4 Departamento de Química y Ciencias Exactas, Sección Química Básica y Aplicada, Universidad Técnica Particular de Loja (UTPL), San Cayetano alto s/n, A.P., 1101608 Loja, Ecuador; 5 Departamento de Ciencias Naturales, Sección de Biología y Genética, Universidad Técnica Particular de Loja (UTPL), San Cayetano alto s/n, A.P., 1101608 Loja, Ecuador

**Keywords:** Parasitology, Medicinal chemistry

## Abstract

Recently, the search for novel therapeutic agents against *Acanthamoeba* species has been focused on the evaluation of natural resources. Among them, marine microorganisms have risen as a source of bioactive compounds with the advantage of the ability to obtain unlimited and constant amounts of the compounds in contrast to other natural sources such as plants. Furthermore, marine actinomycetes have recently been reported as highly rich in bioactive agents including salinosporamides, xiamycines, indolocarbazoles, naphtyridines, phenols, dilactones such as antimycines and macrolides among others. In this study, staurosporine (STS) was isolated from a strain of *Streptomyces sanyensis* and tested against *Acanthamoeba* to characterize the therapeutic potential of STS against this protozoan parasite. We have established that STS is active against both stages of the *Acanthamoeba* life cycle, by the activation of Programmed Cell Death via the mitochondrial pathway of the trophozoite. We have also established that STS has relatively low toxicity towards a macrophage cell line. However, previous studies have highlighted higher toxicity levels induced on other vertebrate cell lines and future research to lower these toxicity issues should be developed.

## Introduction

*Acanthamoeba* genus belongs to the Free-Living Amoebae (FLA) group and includes some strains which are able to cause opportunistic infections in humans and other animals such as encephalitis and keratitis^1^. Current therapeutic approaches against *Acanthamoeba* infections involve the use of a combination of drugs which is highly toxic for the patient and not fully effective. In the case of *Acanthamoeba* Keratitis (AK), most of the commonly used topical agents such as biguanides are used to treat this pathogen^1,2^ but these compounds are unfortunately toxic to human corneal cells. Other compounds used against AK have shown activity but are not available worldwide, hence the problem of treatment continues.

In addition to the therapeutic issues mentioned above, *Acanthamoeba* is able to form a highly resistant cyst stage which complicates the development of fully effective therapeutic agents against this pathogenic protozoa^3–5^.

The search for novel compounds against *Acanthamoeba* species has been lately focused in the evaluation of natural sources presenting anti-*Acanthamoeba* activity^2,6,7^. Among these sources, recent reports have focused on bioactive compounds from marine microorganisms. Mangrove ecosystems in particular, have attracted recently the attention of the scientific community due to the discovery of new drugs with pharmaceutical potential. Mangroves are singular ecosystems, characterized for their high biodiversity and the feasibility to collect samples. More than 200 endophytic fungi and over 2000 actinomycetes have been collected from mangroves, from which a wide array of identified secondary metabolites have displayed a variety of pharmacological properties including antimicrobial, anticancer and antiviral ones among others^8^.

**Figure 1:**
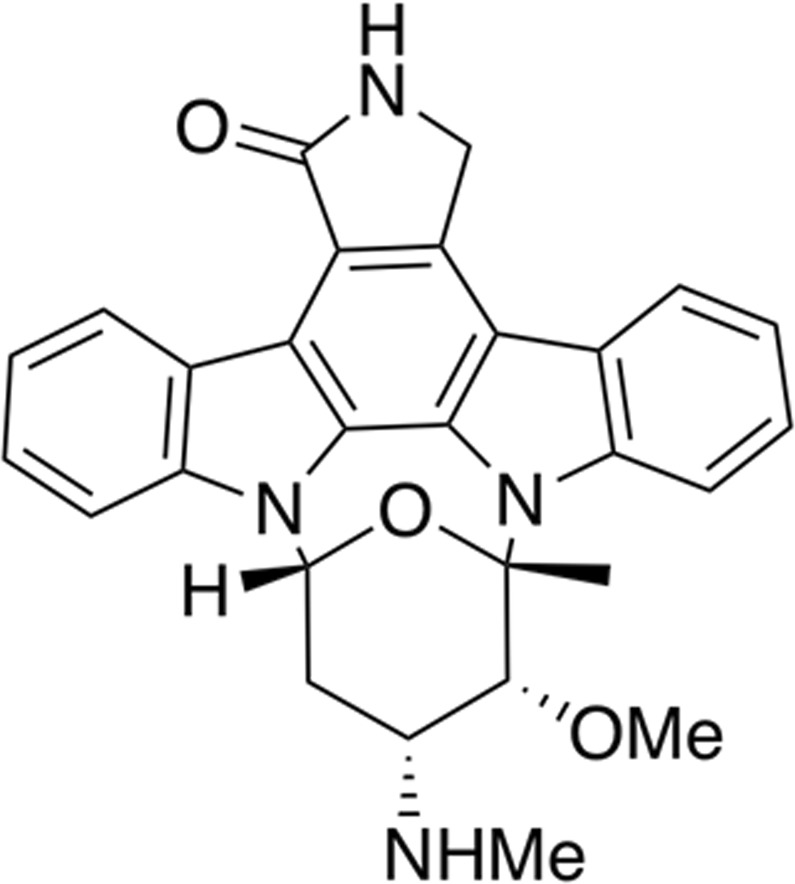
Structure of STS.

Many secondary metabolites have been isolated from marine actynomycetes such as salinosporamides, xiamycines, indolocarbazoles, naphtyridines, phenols, dilactones such as antimycines, macrolides, etc^9,10^. Among actinomycetes, the genus *Streptomyces* has gained particular attention in this research field for its richness in bioactive molecules. In particular, the species *Streptomyces sanyensis*, isolated from a mangrove sediment^11^, was first identified in 2011. Cloning and chemical studies confirmed *S*. *sanyensis* as a prolific producer of indolocarbazole (ICZ) alcaloids^12,13^. The large family of ICZs is characterized by their strong inhibitory effect against many cancer cell lines, platelet aggregation inhibitor, antibacterial, antiviral and neuroprotective properties^14,15^. Staurosporine (STS, Fig. 1), isolated from *Streptomyces staurosporeus*^14^ in 1977, was the first reported ICZ. In addition, STS is a potent affinity inhibitor of protein kinases which blocks the ATP-binding site of the enzimes^16^ and it has been demonstrated that STS induces apoptosis by activation of caspase-3^17^ in higher eukaryotes.

In the present work, we have identified and developed cultures of the strain *Streptomyces sanyensis* PBLC04, a high-yield producer of STS, and tested the activity of this alkaloid against *Acanthamoeba* genus with the aim to characterize its therapeutic potential.

## Results

### Culture and molecular identification of *Streptomyces* sp. PBL04

**Figure 2:**
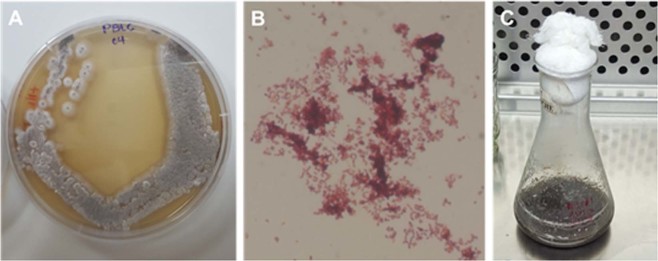
Morphological characteristics on plate and under microscopy (**A**,**B**) as well, metabolic production observed (**C**) on a seawater-based media, from *Streptomyces* sp. PBLC04.

The strain *Streptomyces* sp. PBLC04 showed a high growth rate in 75% seawater-based media, with concave and dry colonies presenting grey aerial mycelia with smooth borders when cultured on plate (Fig. 2). Microscopic examination revealed pseudomycelia and gram-positive rod-shaped bacteria and supported the identification at genus level. Metabolic production of the strain was detected after 7 days of cultivation at 30 °C, 120 rpm, in a flask containing 75% seawater-based media. DNA sequencing of the obtained PCR fragments of *Streptomyces* sp. PBLC04, yielded two sequences which showed 100% similarity, for this reason only one was included in the phylogenetic analysis (Genbank Accession Number MK639686). Phylogenetical analyses demonstrated that *Streptomyces* sp. PBLC04 was included in one clade with bootstrap values 100 NJ/87 ML containing the type sequence 219820 of *Streptomyces sanyensis*. The pairwise distance for both sequences corresponds to 0.37%. The sequences from Strain PBLC04 compared with the other sequences into this clade corresponded to ≤0.22% (1360 bp base pair compared). The alignments used can be obtained from TreeBASE (http://www.treebase.org/) under accession number S24696.

### Isolation and characterization of STS from *Streptomyces sanyensis* PBLC04

Culture of *Streptomyces sanyensis* PBLC04 produced STS in great amounts (134 mg), which represents 1.06% of the total content of the biomass extract. The isolation of STS was carried out through three chromatographic steps which involved: gel-filtration on Sephadex LH-20, flash reversed phase C18 with 5 mM ammonium acetate (NH_4_OAc) buffer at pH 6.0 and Si-gel chromatography. Pure STS was separated and crystallized (28.4 mg) as yellow needles from dichloromethane (DCM).

(+)-Staurosporine possesses the molecular formula C_28_H_24_N_4_O_3_ as determined from the HRESIMS peak found at *m/z* 489.1908 (calc. for 489.1897 [M + Na]^+^), and [α]_D_^20^ + 43 (*c* 0.3, CHCl_3_). Its UV spectrum (CHCl_3_) λ_max_ (log ε) 297 (4.48) nm, showed characteristic peaks of the ICZ chromophore^18^. The IR absorption bands at 2925, 2360, 2341, 1681, 1455, 1353 and 1318 cm^−1^ agreed the presence of an aromatic nucleus, a carbonyl group belonging to the indolocarbazole and a lactam ring^18^. Comparison of ^1^H and ^13^C NMR spectral data (see supplementary material) with those previously reported confirmed the structure of STS^19,20^.

### STS eliminates *Acanthamoeba* trophozoites and cysts at low concentrations and presents low cytotoxicity levels

**Figure 3:**
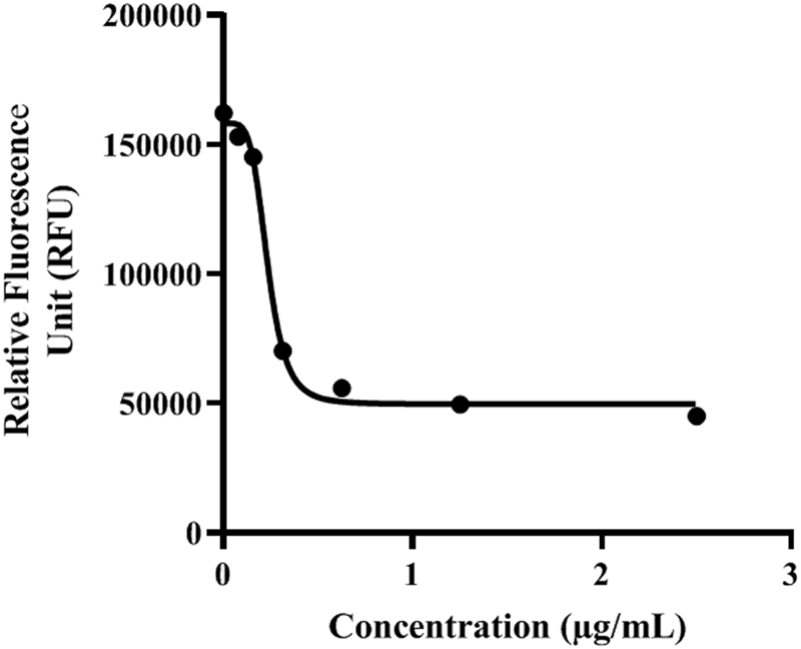
Standard curve analysis for IC_50_ and IC_90_ calculation using increasing doses of STS determined by the Alamar Blue assay.

**Figure 4:**
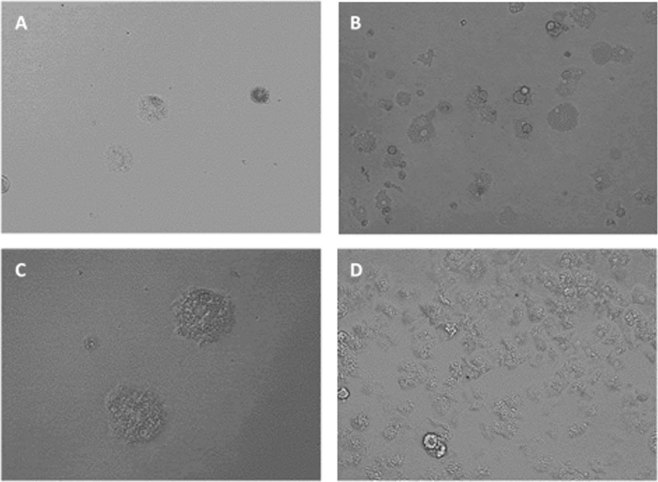
*Acanthamoeba castellanii* Neff trophozoite incubated with various concentrations of STS: 2.5 µg/ml (5.3 µM) (**A** 20X, **C** 40X) and 1.25 µg/ml (2.6 µM) (**B** 20X). Images are representative of the cell population observed in the performed experiments. Negative control (Amoebae alone) (**D** 20X) Images were obtained using an EVOS FL Cell Imaging System AMF4300, Life Technologies, USA. *Acanthamoeba castellanii* Neff cysts incubated with 25 µg/ml (53.5 µM) of STS (**A**), 3.125 µg/ml (6.7 µM) (**B**) and 0.781 µg/ml (1.6 µM) (**C**) compared to the negative control (**D**). Images (20X) are representative of the cell population observed in the performed experiments. Images were obtained using an EVOS FL Cell Imaging System AMF4300, Life Technologies, USA.

Staurosporine was able to kill *Acanthamoeba* trophozoites when incubated *in vitro* in a dose dependent way. The obtained results using the mentioned colorimetric assay in the material and methods section allowed us to establish IC_50_ and IC_90_ values of 0.265 ± 0.057 and 1.27 ± 0,007 µg/mL (0.568 ± 0.122 µM and 2.70 ± 0.015 µM) respectively (Fig. 3). Interestingly, the effects of this compound against the trophozoite stage were seen even at 15 min post incubation with the drug (Fig. 4).

**Figure 5:**
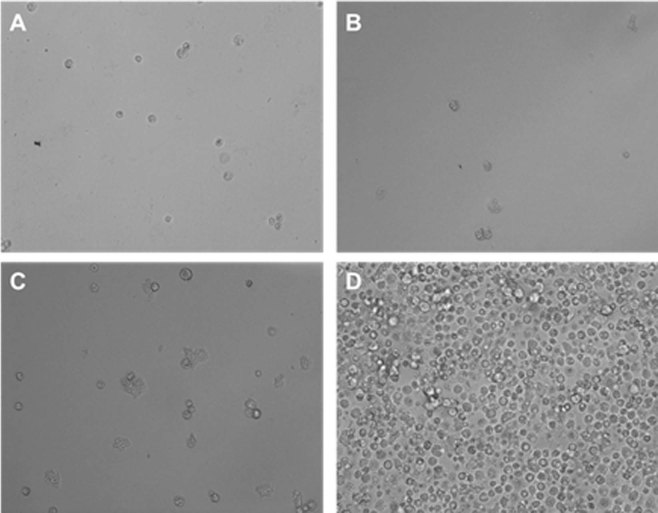
*Acanthamoeba castellanii* Neff cysts incubated with 25 µg/ml (53.5 µM) of STS (**A**), 3.125 µg/ml (6.7 µM) (**B**) and 0.781 µg/ml (1.6 µM) (**C**) compared to the negative control (**D**). Images (20X) are representative of the cell population observed in the performed experiments. Images were obtained using an EVOS FL Cell Imaging System AMF4300, Life Technologies, USA.

In the case of cysticidal activity, STS was also active at low concentrations and dose-dependent, being the calculated IC_50_ of 0.771 ± 0.008 µg/mL (1.653 ± 0.017 µM) (Fig. 5).

**Figure 6:**
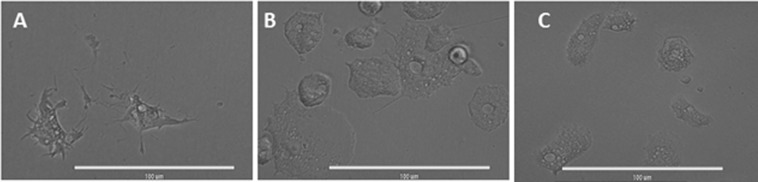
Observed morphological changes in *Acanthamoeba castellanii* Neff trophozoites incubated with 20 µg/ml (42.8 µM) of STS at 15 min (**A**) and 1 h (**B**) compared to the negative control (Amoebae alone) (**C**). Images are representative of the cell population observed in the performed experiments. Images were obtained using an EVOS FL Cell Imaging System AMF4300, Life Technologies, USA (40X). *Acanthamoeba castellanii* Neff trophozoites incubated with IC_90_ of STS and the evolution of chromatin condensation observed for 30 min, 24 h, 48 h, 72 h and 96 h. Hoechst stain is different in control cells, where uniformly faint-blue nuclei are observed, and in treated cells, where the nuclei are bright blue. Red fluorescence corresponds to the propidium iodide stain. Images (10X) are showing chromatin condensation (blue) in *Acanthamoeba* treated cells. Images (10X) are representative of the cell population observed in the performed experiments. Images were obtained using an EVOS FL Cell Imaging System AMF4300, Life Technologies, USA.

Moreover, amoebic forms were observed to present dramatic morphological changes such enlargement of the cytoplasm and loss of cellular structure (lack of amoeboid form), again after 15 min of incubation with the compound (Fig. 6).

Furthermore, toxicity assays against the murine macrophage J774.A1 cell line yielded an CC_50_ of 4.076 ± 0.335 (8.737 ± 0.718 µM). Therefore, the toxicity was considered low when compared to the obtained *Acanthamoeba* inhibitory concentrations of STS.

### STS treated amoebae stained positive in the double stain assay

**Figure 7:**
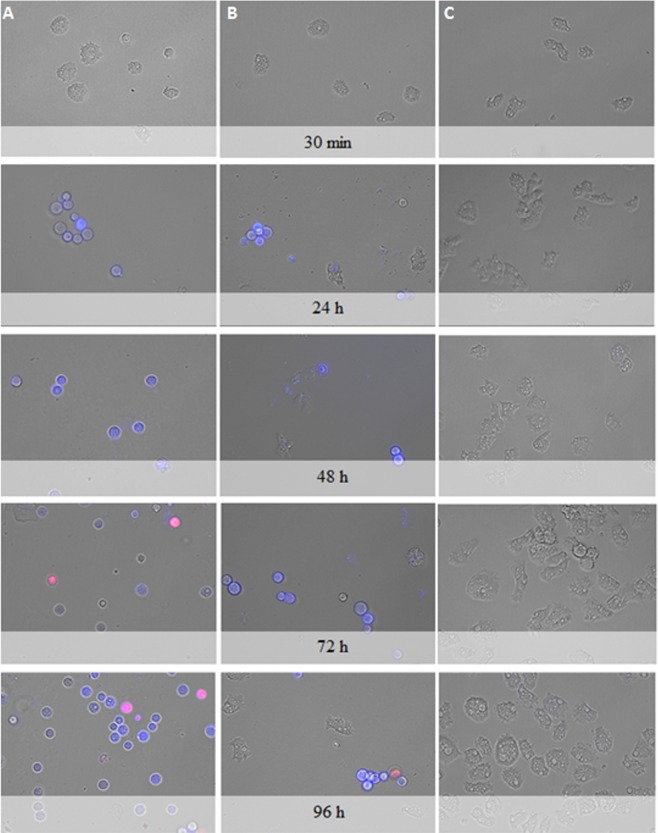
*Acanthamoeba castellanii* Neff trophozoites incubated with IC_50_ (**B**) and IC_90_ (**A**) of STS and the evolution of chromatin condensation observed for 30 min, 24 h, 48 h, 72 h and 96 h. Hoechst stain is different in control cells (**C**), where uniformly faint-blue nuclei are observed, and in treated cells, where the nuclei are bright blue. Red fluorescence corresponds to the propidium iodide stain. Images (40X) are showing chromatin condensation (blue) in *Acanthamoeba* treated cells. Images (40X) are representative of the cell population observed in the performed experiments. Images were obtained using an EVOS FL Cell Imaging System AMF4300, Life Technologies, USA.

After performance of the double staining protocol, it was observed that STS at both concentrations of IC_50_ and IC_90_ could induce chromatin condensation. Treated amoebae showed bright-blue stained nuclei even at 24 h after incubation with the compound as shown in Fig. 7.

### STS caused plasma membrane permeability in treated cells

**Figure 8:**
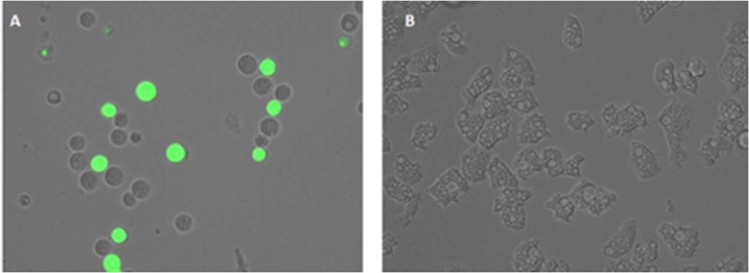
Permeation of the *Acanthamoeba* Neff plasma membrane to the vital dye SYTOX green caused by addition of STS IC_90_ after 24 h (**A**). Negative Control (**B**). Images (40X) are representative of the cell population observed in the performed experiments. Images were obtained using an EVOS FL Cell Imaging System AMF4300, Life Technologies, USA.

Amoebae treated with the IC_90_ of STS induced plasmatic membrane damage after 24 h of incubation as illustrated in Fig. 8. Moreover, it is also important to mention that even though the membrane was damaged, cell integrity was maintained.

### STS induced amoebic mitochondrial malfunction

**Figure 9:**
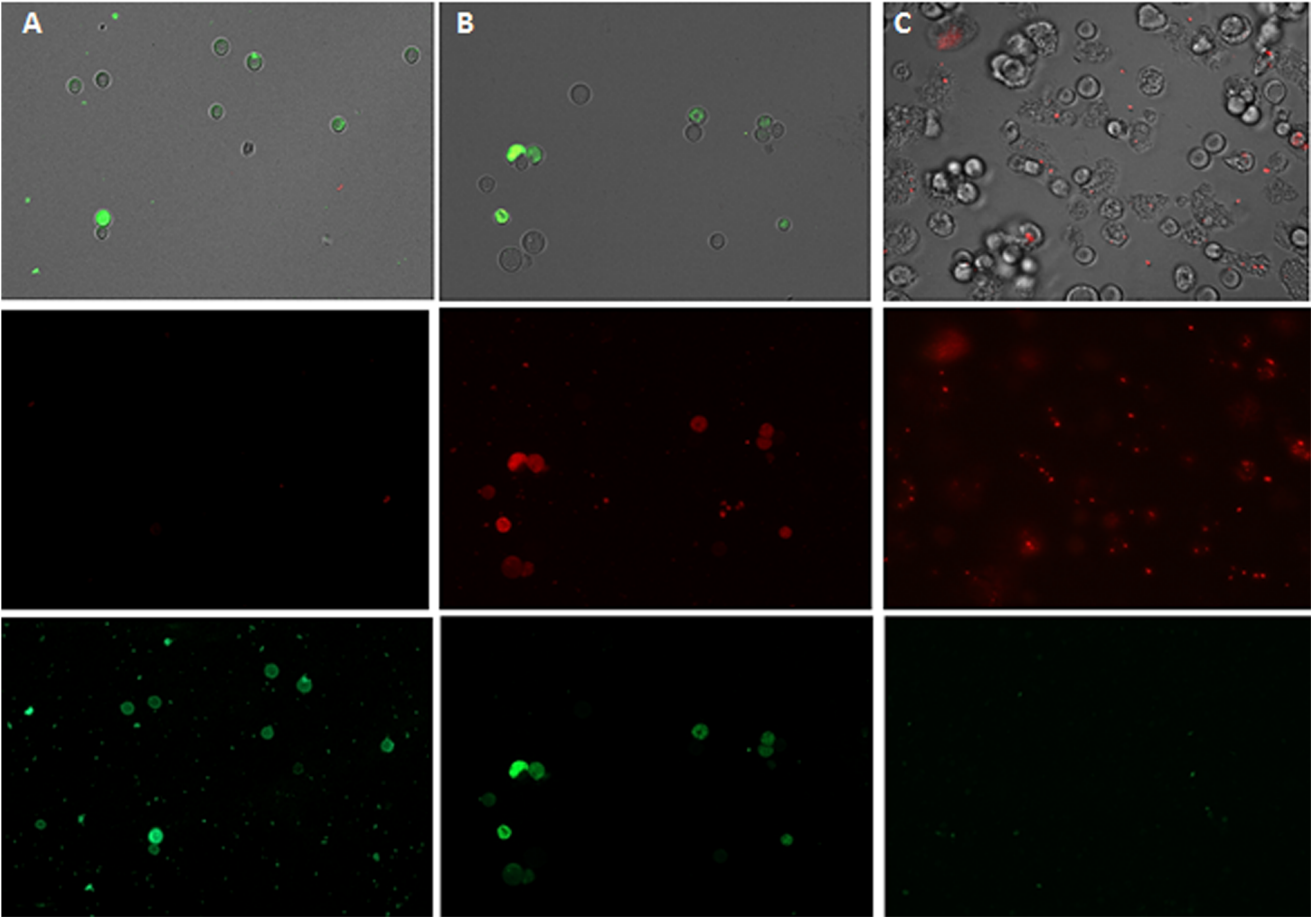
The effect of IC_50_ (**B**) and IC_90_ (**A**) on the mitochondrial potential, JC-1 dye accumulates in the mitochondria of healthy cells as aggregates (**C**) (red fluorescence) in cells treated with the IC_90_ of STS for 24 h, due to collapse of mitochondrial potential, the JC-1 dye remained in the cytoplasm in its monomeric form, green fluorescence. (Images are representative of the population of treated amoeba 20X).

**Figure 10:**
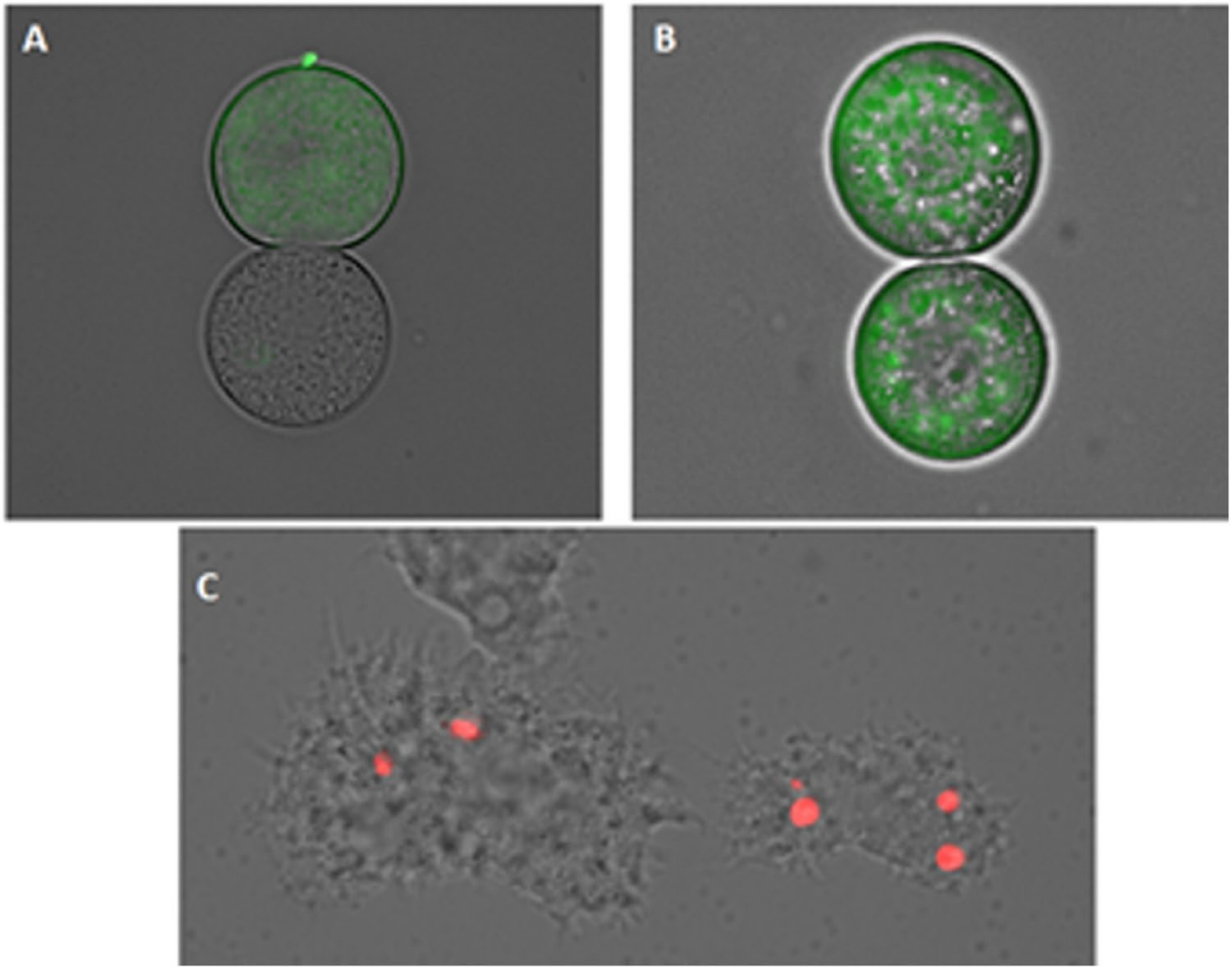
The effect of IC_50_ (**A**) and IC_90_ (**B**) on the mitochondrial potential observed with a higher magnification 100X. (**C**) Negative control: cells without any treatment.

Figures 9 and 10, show STS induced changes on the mitochondrial potential since the JC-1 dye remained in the cytoplasm in its monomeric form, shown as green fluorescence. Furthermore, the mitochondrial damage was also checked by measuring the ATP level generated in 24 h. The observed results showed that STS IC_90_ treated cells presented a 20% decrease of ATP levels when compared to untreated cells.

### STS increases Reactive Oxygen Species (ROS) levels in *Acanthamoeba*

**Figure 11:**
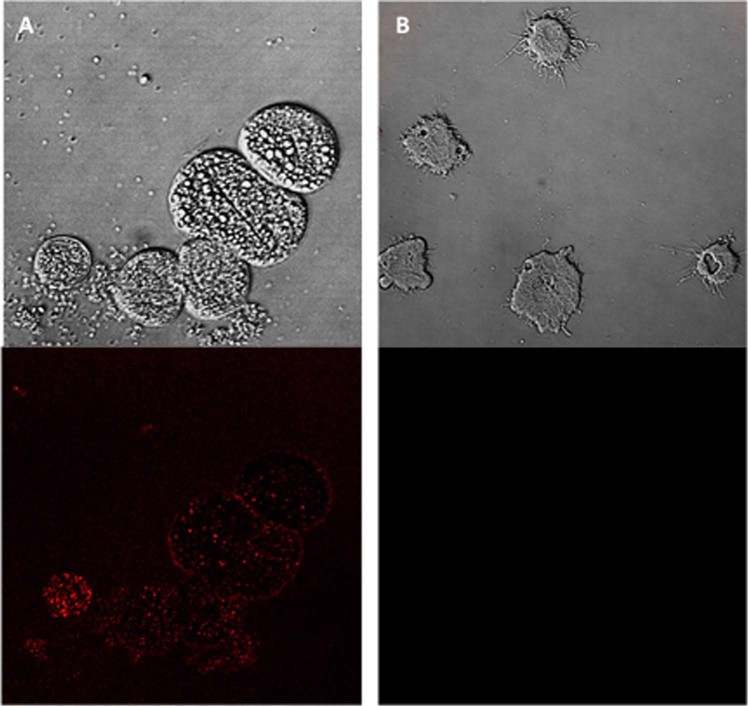
Increased levels of ROS in *Acanthamoeba* Neff caused by addition of STS IC_90_ after 24 h (**A**). Negative Control (**B**) cells exposed to CellROX Deep Red in absence of STS. Images (63X) are representative of the cell population observed in the performed experiments. Cells were observed in a Leica TSC SPE- confocal microscope.

STS treated amoebae caused increased levels of ROS when treated with IC_90_ after 24 h of incubation (Fig. 11).

## Discussion

Marine microorganisms, in particular actinobacteria, are responsible for the production of approximately half of the known bioactive secondary metabolites: mainly antibiotics, antitumor agents, and immunosuppressants^21^. The biotechnological potential of marine microorganisms provides the additional advantage in providing a continuous supply of the compound of interest, and to avoid drawbacks such as seasonal occurrence of metabolites, usually linked to other natural sources such as seaweeds or marine invertebrates. In the present study, the strain *Streptomyces sanyensis* PBLC04, isolated from a mangroove ecosystem in Ecuador, has allowed us the isolation of STS as the major compound when cultured under laboratory conditions. Although STS was first identified and isolated from a strain of *S*. *staurosporeus in* 1977^14^, to the best of our knowledge, this is the first time that this molecule is assayed against *Acanthamoeba* genus.

Regarding the obtained activities against both the trophozoite and cyst stage of *Acanthamoeba*, it is important to highlight that STS was active at low concentrations when compared to other commonly used drugs against *Acanthamoeba* such as chlorhexidine (IC_50_ = 1.10 ± 0.01 µM) amphotericin B (IC_50_ = 65.98 ± 5.91 µM) or PHMB (0.02%)^22,23^. Moreover, as it was mentioned in the results section, toxicity values when tested against a macrophage cell line were low in comparison to the obtained IC_50_ and IC_90_ with values 4-fold higher than the STS IC_50_ against cysts. However, previous research has shown higher toxicity values after 12 h of exposure to STS^24–26^.

Programmed Cell Death (PCD) has been previously reported in our laboratory in *Acanthamoeba* genus after incubation with compounds such as statins and voriconazole among other molecules^2,27,28^. The process of PCD involves multiple changes such as chromatin condensation, cell shrinkage and loss of mitochondrial potential among other phenomena^29^.

**Figure 12:**
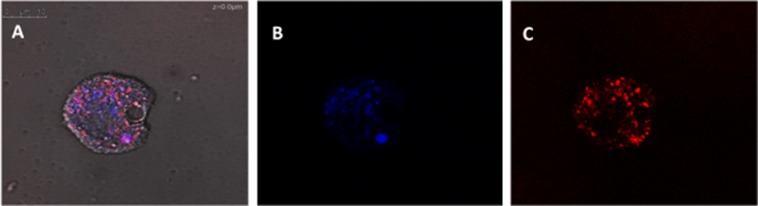
*Acanthamoeba castellaniii* Neff trophozoite (63X) stained with Hoechst and CellROX Deep Red after 24 h incubation of amoebae with IC_90_ of staurosporine (**A**). A bright condensed nucleus is observed as well as bright blue staining. Moreover, red dots mark regions of the cytoplasm where ROS species were generating. Blue channel for Hoechst (**B**). Red channel for CellROX Deep Red (**C**). Cells were observed in a Leica TSC SPE- confocal microscope.

In the present study, STS was shown to induce plasma membrane damage, chromatin condensation, collapse of ATP level and mitochondrial membrane potential as well as increased levels of ROS. Since these effects were observed even after 15 min of incubation with the compound, it is probable that STS induces damages at the cellular membrane level, entering the cytoplasm without necrotic effects. We argue STS induces apoptosis in *Acanthamoeba* through the intrinsic pathway since the mitochondrial potential was collapsed even at 24 h (as well as ATP levels) (Fig. 12). PCD is also indicated by the generation of ROS species and condensation of DNA.

The family of proteins kinases are considered to be the major target of STS. Furthermore, this molecule has demonstrated antiproliferative activity in several human cancer cell lines, inducing apoptosis by activation of caspase-3^30^. These findings in higher eukaryotic cells support the conclusions of our study. The genome of this amoeba^31^ is known to be rich in kinase genes and this may be the reason why it is so sensitive.

## Conclusion

In conclusion, *Streptomyces sanyensis* strain PBLC04 produces STS as the major metabolite. Evaluation of the activity of this compound against *Acanthamoeba* trophozoites and cysts, have shown a high activity of this compound. Moreover, STS-treated amoebae started apoptosis via the mitochondrial pathway. Therefore, STS is presented as a novel, highly active, PCD inducer and low toxic anti-*Acanthamoeba* compound at least *in vitro* against the used macrophage cell line which should be exploited for the development of novel therapeutic agents. However, due to the observed toxicity levels in previous studies using other cell lines such as human endothelial cell lines and even the eye, future research should focus on the development of improved delivery of STS in the host tissue which allow to lower toxicity.

## Methods

### General experimental procedures

Optical rotations were measured in CH_2_Cl_2_ on a PerkinElmer 241 polarimeter by using a Na lamp. NMR spectra were recorded on a Bruker AVANCE 600 MHz instrument equipped with a 5 mm TCI inverse detection cryoprobe. NMR spectra were obtained dissolving samples in CD_2_Cl_2_ (99.9%) and chemical shifts are reported relative to solvent (δ_H_ 5.32 and δ_C_ 54.0 ppm). Standard Bruker NMR pulse sequences were utilized. HR-ESI-MS data were obtained on an LCT Premier XE Micromass spectrometer. IR spectra were recorded on a Perkin-Elmer Spectrum BX spectrometer. UV spectra was recorded in a Jasco V-560 UV/Vis spectrophotometer. EnSpire Multimode Reader (Perkin Elmer) using absorbance values of Alamar Blue reagent. TLC (Thin layer chromatography) (was visualized by UV light (254 and 365 nm).

### Materials

Ammonium acetate (NH_4_OAc, LiChropur, Merck, Germany), Methylene chloride-*d*2 (Euriso-top, 99.90%D, UK), Methanol-*d*4 (water < 0.03%, euriso-top), RP18-prepacked cartridge (25–40 µm 70 g, Götec-Labortechnik GmbH), Sephadex LH-20 (Sigma-Aldrich), Starch, yeast extract and Proteose peptone from DIFCO, Calcium carbonate (Merck), Potassium bromide (Merck), Ferric sulfate (Sigma-Aldrich), TLC plates Silica gel G60 F254-Merck.

### Collection and characterization of *Streptomyces sanyensis* PBLC04 strain

*Streptomyces sanyensis* PBLC04 strain was collected in Jambelí mangrove (3°15′792″S, 80°00′739″W - 03°17′711″S, 80°01′924″W), Ecuador. The strain is part of the microbial collection of Universidad Técnica Particular de Loja (UTPL, Loja-Ecuador) and the methodology for extraction, culturing and isolation of pure strains from mangrove sediments was performed as described by Cartuche L. *et al*.^32^.

### DNA isolation, PCR and phylogenetic analysis

The DNA was extracted with PureLink Genomic DNA Mini Kit (Invitrogen) for gram-positive bacteria, according to manufacturing specifications. Partial 16S rDNA region was amplified by using the universal primers for bacteria: 27F (5′-AGAGTTTGATCMTGGCTCAG-3′) and 1492R (I) (5′-GGTTACCTTGTTACGACTT-3′)^33^. The PCR protocol used was: initial denaturation at 98 °C for 30 s, followed by 30 cycles of 98 °C denaturation for 10 s, annealing at 60 °C for 20 s, extension at 72 °C for 30 s and a final extension at 72 °C for 10 min. The volume of reaction was 20 μL, 1 ρmol each primer and 1U of Finnzymes’ Phusion High-Fidelity DNA Polymerase of final concentration. PCR products were visualized on 1% (w/v) agarose gels stained with 5% (v/v) GelRed (Biotium, Hayward, USA). The purified products were sequenced at Macrogen (Seoul – Republic of Korea).

The sequence chromatograms were verified using the software Codon Code Aligner 5.1.4 (CodonCode Corporation, Centerville, MA, USA). The obtained sequences were aligned with sequences available at the International Nucleotide Sequence Database Collaboration (INSDC) (http://www.insdc.org/). All 16S rDNA sequences were aligned using the G-INS-i strategy as implemented in MAFFT V7^34^. Three phylogenetic trees were built using a Neighbour-Joining (NJ) analysis with BIONJ modification of the NJ algorithm^35^, Maximum Likelihood (ML) based on 1000 bootstrap replications^36^ and GTRMIX as DNA substitution model^37^ implemented in MEGA v.7 software^38^.

### Fermentation conditions and culture extraction

*Streptomyces sanyensis* PBLC04 strain was cultured in 30 L of a modified seawater-based medium (A1) consisting of 75% seawater containing 10 g starch, 4 g yeast extract, 2 g proteose peptone, 1 g calcium carbonate, supplemented with 5 mL/L of a solution of potassium bromide (67 mM) and ferric sulfate (20 mM)^39^. Fernbach flasks with 1 L each were kept at 30 °C and mixed in an orbital shaker at 200 rpm for 7 days.

Cultures of *S*. *sanyensis* PBLC04 were centrifuged at 5000 rpm for 10 min to separate supernatant from cell biomass. Amberlite XAD7-HP resin (20 g/L) were added to the supernatant and stirred for 3 h to adsorb the excreted metabolites. After filtration, both the resin and the biomass pellet were separately macerated with a mixture of MeOH:AcOEt:Acetone (2:7:3) for 12 h at 120 rpm. Then, the two resulting extracts were filtered and dried at reduced pressure at 30 °C in a rotary evaporator.

### Isolation of STS from biomass extract

The salt-free biomass extract (12.6 g) was fractionated by size-exclusion chromatography on Sephadex-LH-20 with methanol as eluent to yield 10 fractions that were finally gathered in four final fractions according to TLC and ^1^H-NMR analysis. Fractions 3 and 4 exhibited characteristic signals for ICZs in the ^1^H-NMR spectrum (MeOH-*d*_4_)^20^. Fraction 4 (716.0 mg) was separated by flash chromatography on a RP18 prepacked cartridge (25–40 µm, 70 g, Götec-Labortechnik GmbH) with a step gradient elution system of H_2_O:MeOH, 5 mM NH_4_OAc, (20% to 100% MeOH) at 4 mL/min flow and UV detection at 254 nm, to afford seven fractions according to TLC and ^1^H-NMR analysis. The active fraction (124.0 mg) was further purified by elution on Si-60 open column (230–400 mesh, 60 Å) using CHCl_3_:MeOH (9:1) as elution system to obtain 106.0 mg. Pure STS was separated and crystallized (28.4 mg) as yellow needles using DCM.

### *In vitro* drug sensitivity assay

#### Strain used

The anti-*Acanthamoeba* activity of STS was evaluated against the *Acanthamoeba castellanii* Neff (ATCC 30010) type strain from the American Type Culture^6,7^. This strain was grown axenically in PYG medium (0.75% (w/v) proteose peptone, 0.75% (w/v) yeast extract and 1.5% (w/v) glucose) containing 40 μg gentamicin ml^−1^ (Biochrom AG, Cultek, Granollers, Barcelona, Spain).

#### In vitro effect against the trophozoite stage of acanthamoeba

The anti-*Acanthamoeba* activities of the tested compound was determined by the Alamar Blue assay as previously described^2,27^. Briefly, *Acanthamoeba* strains were seeded in duplicate on a 96-well microtiter plate with 50 μL from a stock solution of 10^4^ cells/mL. Amoebae were allowed to adhere for 15 min and 50 μL of serial dilution series of the tested compound was added. Finally, the Alamar Blue Assay Reagent (Bioresource, Europe, Nivelles, Belgium) was added into each well at an amount equal to 10% of the medium volume. The plates were then incubated for 96 h at 28 °C with a slight agitation and the emitted fluorescence was examined with an Enspire microplate reader (PerkinElmer, Massachusetts, USA) at 570/585 nm.

#### In vitro effect against the cyst stage of acanthamoeba

The cysticidal activity of STS was determined by the Alamar Blue assay and confirmed visually by inverted microscopy. Cysts of *A*. *castellaniii* Neff were prepared as previously described^40^. Briefly, trophozoites were transferred from PYG medium based cultures (trophozoite medium) to Neff´s encystment medium (NEM; 0.1 M KCl, 8 mM MgSO_4_·7H_2_O, 0.4 mM CaCl_2_·2H_2_O, 1 mM NaHCO_3_, 20 mM ammediol [2-amino-2-methyl-1,3-propanediol; Sigma Aldrich Chemistry Ltd., Madrid, Spain], pH 8.8, at 25 °C) and were cultured in this medium with gently shaking for a week in order to obtain mature cysts. After that, mature cysts were harvested and washed twice using PYG medium.

A serial dilution of the tested molecules was made in PYG. The *in vitro* susceptibility assay was performed in sterile 96-well microtiter plates (Corning™). To these wells, containing 50 µL of drug dilutions, 5·10^4^ mature cysts of *Acanthamoeba*/mL were added. After 7 days of incubation with the drugs, the plate was centrifuged at 3000 rpm for 10 min. The supernatant was removed and replaced with 100 µL of fresh medium PYG in each well. Finally, 10 μL of the Alamar Blue Assay Reagent (Biosource, Europe, Nivelles, Belgium) was placed into each well, and the plates were then incubated for 144 h at 28 °C and the emitted fluorescence was periodically examined with an Enspire microplate reader (PerkinElmer, Massachusetts, USA) at 570/585 nm.

#### Cytotoxicity assays

Cytotoxicity of STS was evaluated after 24 h incubation of murine macrophage J774.A1 cell line (ATCC # TIB-67) with different concentration of the tested compound at 37 °C in a 5% CO_2_ humidified incubator. The viability of the macrophages was determined with the Alamar Blue assay as previously described^2^.

#### Double-stain assay for programmed cell death determination

A double-stain apoptosis detection kit (Hoechst 33342/PI) (GenScript, Piscataway, NJ, USA) and an EVOS FL Cell Imaging System AMF4300, Life Technologies, USA were used. The experiment was carried out by following the manufacturer’s recommendations, and 10^5^ cells/well were incubated in a 24-well plate for 24 h with the previously calculated IC_90_. The double-staining pattern allows the identification of three groups in a cellular population: live cells will show only a low level of fluorescence, cells undergoing PCD will show a higher level of blue fluorescence (as chromatin condenses), and dead cells will show low-blue and high-red fluorescence (as the Propidium Iodide stain enters the nucleus).

### Intracellular ROS production using CellROX deep red staining

The generation of intracellular ROS was detected using the CellROX Deep Red fluorescent probe (Invitrogen). The cells were treated with IC_90_ of STS for 24 h and exposed to CellROX Deep Red (5 μM, 30 min) at 26 °C in the dark. Cells were observed in a Leica TSC SPE- confocal microscope equipped with inverted optics at λ_exc_ = 633 and λ_em_ = 519 nm.

#### Analysis of mitochondrial membrane potential

The collapse of an electrochemical gradient across the mitochondrial membrane during apoptosis was detected with the JC-1 mitochondrial membrane potential detection kit (Cell Technology. After being treated with IC_90_ of the test solution for 24 h, the cells were centrifuged (1000 r.p.m. ×10 min) and resuspended in JC-1 buffer. Images were taken on an EVOS FL Cell Imaging System AMF4300, Life Technologies, USA from Life Technologies (Madrid, Spain). The staining pattern allows the identification of two groups in a cellular population: live cells will show only red fluorescence; cells with low mitochondrial potential, (undergoing PCD) will show a higher level of green and red fluorescence.

#### Measurement of ATP levels

ATP level was measured using a CellTiter-Glo Luminescent Cell Viability Assay. The effect of the drug on the ATP production was evaluated by incubating (10^5^) of cells/ml with the previously calculated IC_50_ and IC_90_ of STS.

#### Plasma membrane permeability

The SYTOX Green assay was performed to detect alterations of the membrane permeability in treated cells. Briefly, 10^5^ trophozoites were washed and incubated in saline solution with the SYTOX Green at a final concentration of 1 μM (Molecular Probes) for 15 min in the dark. Subsequently STS solution was added (IC_90_). After 24 h of treatment, cells were observed in a Leica TSC SPE- confocal microscope equipped with inverted optics at λ_exc_ = 482 nm and λ_em_ = 519 nm^9^.
